# Inverse correlation between serum irisin and cardiovascular risk factors among Chinese overweight/obese population

**DOI:** 10.1186/s12872-021-02380-0

**Published:** 2021-11-30

**Authors:** Ruoyi Liu, Qiao Zhang, Nianchun Peng, Shujing Xu, Miao Zhang, Ying Hu, Zhengyi Chen, Kun Tang, Xi He, Yi Li, Lixin Shi

**Affiliations:** 1 Department of Endocrinology and Metabolism, Affiliated Hospital of Guizhou Medical University, No. 28 Guiyi Road, Yunyan District, Guiyang, 550001 China; 2 Present Address: Department of Endocrinology and Metabolism, Guizhou Provincial People’s Hospital, Guiyang, 550001 China

**Keywords:** Irisin, Cardiovascular disease, Diabetes mellitus, Hypertension, Dyslipidemia

## Abstract

**Background:**

Irisin is a novel myokine associated with obesity, which is a traditional cardiovascular risk factor (CVRF). The present study aimed to investigate the association between serum irisin and a single CVRF as well as the clustering of CVRFs among Chinese overweight/obese population.

**Methods:**

A total of 98 overweight and 93 obese subjects without clinical treatments were enrolled in this study. Subjects were then divided into two groups, based on the serum irisin level: a low irisin group (1.10–13.44 ng/ml) and a high irisin group (13.49–29.9 ng/ml). The clustering of CVRFs, smoking, diabetes mellitus, dyslipidemia and hypertension, was classified as 0, 1, 2 and ≥ 3 CVRFs. The demographic and baseline clinical characteristics of all participants were collected and serum irisin was measured.

**Results:**

The high serum irisin group had significantly higher high-density lipoprotein cholesterol but lower fasting plasma glucose than the low serum irisin group. Additionally, the high serum irisin group had a significantly lower prevalence of smoking, diabetes mellitus and dyslipidemia than the low serum irisin group. Increased serum irisin was significantly associated with a reduced risk of smoking and dyslipidemia in both the unadjusted and adjusted models. Furthermore, high serum irisin significantly reduced the risk of the prevalence of 1, 2 and ≥ 3 CVRFs.

**Conclusions:**

among the Chinese overweight/obese populations, high serum irisin is negatively associated with smoking, dyslipidemia and the clustering of CVRFs. Thus, high serum irisin is potentially associated with a low risk of cardiovascular diseases in the Chinese overweight/obese population.

## Background

In modern society, a chronic high-calorie diet and a sedentary lifestyle has resulted in obesity becoming a worldwide epidemic issue [1]. Obesity-related cardiovascular risk factors (CVRFs), such as diabetes mellitus, hypertension and dyslipidemia, have promoted the development and progression of atherosclerosis, which contributes to the leading cause of death associated with cardiovascular diseases globally [2, 3]. It has been noted that the clustering of CVRFs in an individual results in a higher risk of the development of cardiovascular diseases than in an individual that carries only a single CVRF [2–4]. Correspondingly, it has been well recognized that reducing CVRFs prevents the occurrence of atherosclerosis and even cardiovascular diseases among the obese population [5, 6] Thus, identification of available biomarkers that are related to CVRFs is imperative to predict and monitor the pathogenesis and progression of cardiovascular diseases.

Irisin is a new myokine that was initially found to be secreted into the blood stream by skeletal muscles during exercise. Irisin can stimulate the browning of white adipose tissue, to improve glucose metabolism and thus increase energy expenditure [7]. More recent studies have shown that other tissues/organs, such as the heart, brain, tongue, rectum and adipose tissue also secret irisin [8–10]. Clinical studies have shown a correlation between the circulating levels of irisin and cardiovascular diseases. For instance, subjects with coronary artery disease or calcification have lower serum irisin levels than in healthy subjects [11–13], and a lower serum irisin concentration was found to be inversely associated with endothelial dysfunction [14]

and cardiovascular complication among diabetic patients [15]. Moreover, a meta analysis involving 7 case–control studies confirmed that irisin levels were significantly lower in patients with coronary artery disease [16].

In addition, a number of studies conducted among Chinese subjects also showed a relationship between circulating irisin levels and CVRFs. For example, elevated serum irisin levels have been linked to the reduced metabolic syndrome and negatively associated with the dysregulated lipid profile [17], while decreased serum irisin levels are associated with type 2 diabetes mellitus or insulin resistance [18]. Hence, serum irisin levels may offer a value in predicting beneficial effects of exercise on metabolism, as well as the risk of the development of cardiovascular diseases, such as acute heart failure [19].

Previous studies have mainly focused on the relationship between serum irisin levels and cardiovascular diseases as well as a single CVRF; the association between serum irisin levels and the clustering of CVRFs has not been well explored. Moreover, whether serum irisin levels could serve as a biomarker for cardiovascular diseases among a Chinese overweight/obese population is unclear. In the present study, we explored the association between serum irisin levels and a single CVRF, as well as the clustering of CVRFs, and interrogated the potential of serum irisin as a biomarker of cardiovascular diseases among a Chinese overweight/obese population.

## Methods

### Study design and subjects’ selection

**Fig. 1:**
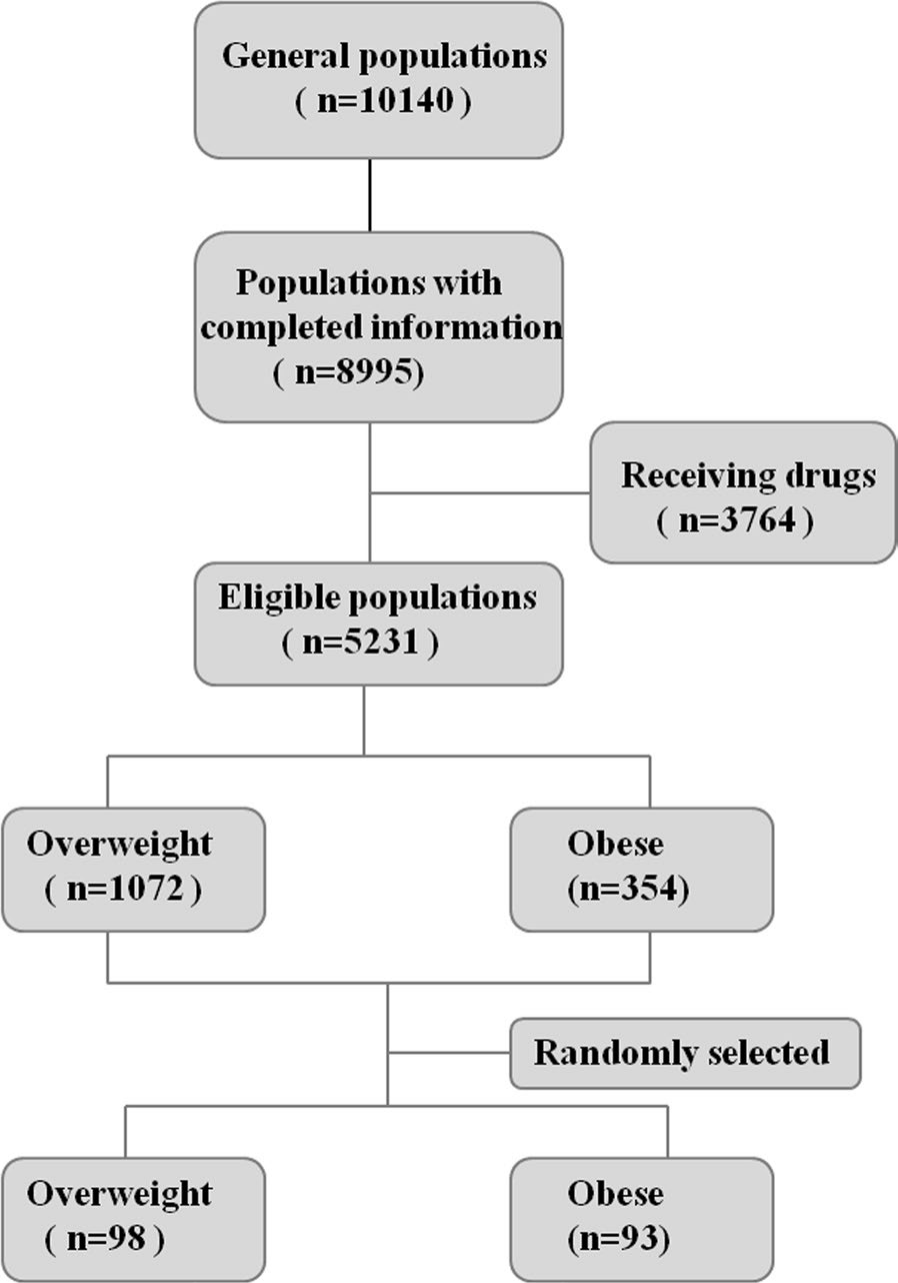
Flow chart showing the participant screening process employed in the present study

We conducted a population-based cross-sectional study in Guiyang, Guizhou, China from May 2011 to August 2011, which was based on Risk Evaluation of cAncers in Chinese diabeTic Individuals: a lONgitudinal study (REACTION) [20]. A total of 10,140 residents aged 40 years or older were initially enrolled. Figure 1 shows the flow chart of the screening process that was employed in the present study. Among these 10,140 subjects, full information was provided for 8995 subjects, and 3764 subjects were excluded from this study due to the receipt of medical treatments, including anti-diabetic, anti-hypertensive and anti-hyperlipidemic drugs, to avoid potential confounders. Finally, 5231 subjects were determined to be eligible for this study. According to the criteria set for overweight/obesity in the Chinese population [21], there were 1072 overweight subjects (with a body mass index [BMI] of 24.0–27.9 kg/m^2^) and 354 obese subjects (BMI ≥ 28 kg/m^2^). In the present study, 98 overweight and 93 obese subjects were respectively randomly selected from these 1072 and 354 subjects. Among all of the 191 enrolled subjects, no one was diagnosed as having coronary artery disease or heart failure. Subjects were then divided into two group by the median value of the serum irisin levels: a low irisin group (1.10–13.44 ng/ml) and a high irisin group (13.49–29.9 ng/ml). The whole study protocol was approved by the Human Research Ethics Committee of the Affiliated Hospital of Guizhou Medical University, and all participants provided written informed consents.

### Data collection and anthropometric measurements

All subjects were asked to complete a standard questionnaire under the guidance of a member of staff who had been given rigorous training for the epidemiologic screening approaches [22]. Demographic characteristics, personal medical history and life styles were recorded through the questionnaire. Active physical activity was defined as performing moderate or robust physical activity for no less than 30 min per day at least 3 days per week, by asking questions on intensity, duration, and frequency of physical activity, which were “How many times a week do you exercise?”, “How long do you exercise per time?”, and “What kind of exercise do you do?” Current smoking was defined according to the self-report, by asking question, which was “Do you smoke now?”. Height, waist circumference (WC) and weight were determined using a calibrated scale. BMI was calculated by dividing the weight (kg) by (height × height) (meters). Blood pressure was measured using an electric sphygmomanometer (OMRON) after at least 15 min of rest. Three blood pressure measurements were made and the average was used as the final read.

### Biochemical measurements

All blood samples from the subjects were obtained after 10 h of fasting, and were frozen at -80℃ for the subsequent analysis. An oral glucose tolerance test (OGTT; 75 g) was conducted for every subject. The concentration of fasting plasma glucose (FPG) and the 2-h plasma glucose after the OGTT (2hPG) were determined using the hexokinase method. The levels of fasting insulin and lipid profile, including the high-density lipoprotein cholesterol (HDL-C), low-density lipoprotein cholesterol (LDL-C), total cholesterol (TC), and triglyceride (TG), were measured using an auto-analyzer (c16000 system, ARCHITECT ci16200 analyzer, Abbott Laboratories, Illinois, USA). Homeostatic model assessment (HOMA)-insulin resistance (IR) was calculated as follows: FPG (mmol/l) × FINS (mU/ml)/22.5. Serum creatinine was measured using an autoanalyser. The serum irisin concentration was measured using an enzyme-linked immunosorbent assay (ELISA) kit (#EK-067-29, Phoenix Pharmaceuticals, USA). This kit detected serum irisin within a range from 0.1 to 1000 ng/ml and with both intra-and inter-assay coefficients of variation less than 10%, which was considered to be the best available ELISA kit for measuring human irisin levels that was on the market [23].

### Study definitions

Based on the American Diabetes Association (ADA) [24], diabetes mellitus was defined as a case in which a 2hPG is ≥ 11.1 mmol/l and/or a FPG of ≥ 7.0 mmol/l. Hypertension was defined as a case in which the average diastolic blood pressure (DBP) ≥ 90 mmHg, the systolic blood pressure (SBP) ≥ 140 mmHg, or both, by the Seventh Report of the Joint National Committee on Prevention, Detection, Evaluation, and Treatment of High Blood Pressure (JNC7) [25]. Dyslipidemia was defined as a case in which at least one of the following measurements was observed: LDL-C ≥ 3.4 mmol/l, HDL-C < 1.0 mmol/l, TG ≥ 1.7 mmol/l, TC ≥ 5.2 mmol/l. In the present study, we evaluated four CVRFs: smoking, diabetes mellitus, hypertension and dyslipidemia. The clustering of CVRFs was classified as 0 CVRF, 1 CVRF, 2 CVRFs and ≥ 3 CVRFs.

### Statistical analyses

All statistical analysis were performed using SPSS 17.0 software. The Kolmogorov–Smirnov test was used to evaluate the normal distribution of continuous variables, which are presented as the mean ± SD (x ± s) and compared using ANOVA and independent t-test between groups. Non-normal variables are presented as the median (interquartile range; M(Q1-Q2)) and were compared using a nonparametric test, such as the Mann–Whitney test. Categorical data are presented as percentages and were compared using the χ^2^ test. Univariable and multivariable linear regression was used to determine the correlation between the serum irisin levels and parameters. Binary and multivariate logistic regression were used to evaluate the odds ratio (OR) of the serum irisin level being associated a single CVRF or clustering CVRFs. Age, gender, active physical activity and other CVRFs are adjusted when estimate OR. Two-sided *p *values < 0.05 were considered statistically significant.

## Results

### Comparison of demographic and baseline clinical characteristics of participants between male and female

The demographic and baseline clinical characteristics of subjects based on gender are shown in Table 1. The male subjects had significantly higher WC, FPG, TG, SBP, DBP, serum creatinine, regular exercise-related physical activities and were more likely a smoker than the female subjects. However, the male subjects had lower HDL-C than the female subjects. Additionally, the female subjects had higher serum irisin levels than the male subjects, with marginal significance.

**Table 1:** Comparison of demographic and baseline clinical characteristics of participants between male and female subjects

	Men	Women	Total	*p* value^a^
N (%)	50 (26.2%)	141 (73.8%)	191	
Age (y)	57.0 ± 8.4	56.6 ± 8.4	56.7 ± 8.4	0.771
Smoking (%)	25 (50.0%)	13 (9.2%)	38 (19.2%)	≤ 0.001**
Active physical activity (%)	31 (62.0%)	44 (31.2%)	75 (39.3%)	≤ 0.001**
BMI (kg/m^2^)	27.7 ± 2.5	27.4 ± 2.4	27.5 ± 2.4	0.588
WC (cm)	93.5 ± 6.5	90.9 ± 7.6	91.6 ± 7.4	0.032*
FPG (mmol/l)	5.9 (5.6, 6.3)	5.6 (5.4, 6.0)	5.7 (5.4, 6.1)	0.003**
2hPG (mmol/l)	7.8 (6.4, 9.8)	7.7 (6.7, 8.9)	7.7 (6.7, 9.2)	0.575
TC (mmol/l)	4.6 ± 1.2	4.7 ± 1.2	4.7 ± 1.2	0.666
TG (mmol/l)	1.6 (1.2, 3.2)	1.5 (1.1, 2.0)	1.6 (1.1. 2.2)	0.043*
LDL-C (mmol/l)	2.7 ± 0.9	2.8 ± 0.8	2.7 ± 0.8	0.919
HDL-C (mmol/l)	1.0 ± 0.2	1.3 ± 0.3	1.2 ± 0.3	≤ 0.001**
SBP (mmHg)	127.2 ± 14.9	121.1 ± 18.2	122.7 ± 17.5	0.036*
DBP (mmHg)	83.5 ± 10.4	78.0 ± 9.0	79.4 ± 10.2	0.001**
Fasting insulin (mIU/l)	10.2 ± 4.5	10.3 ± 3.8	10.3 ± 4.0	0.879
HOMA-IR	2.7 (1.9, 3.4)	2.6 (2.0, 3.3)	2.6 (1.9, 3.3)	0.857
Serum creatinine (mmol/l)	84.0 ± 14.4	63.2 ± 10.6	68.7 ± 14.9	≤ 0.001**
Serum irisin (ng/ml)	12.97 ± 4.40	14.44 ± 4.74	14.05 ± 4.68	0.051

Continuous data are presented as mean ± SD (x ± s) or median (interquartile range; M(Q1-Q2)). Categorical data are presented as percentages

*BMI* body mass index, *HOMA-IR* homeostatic model assessment-insulin resistance, *WC* waist circumference, *FPG* fasting plasma glucose, *2hPG* 2 h plasma glucose, *SBP* systolic blood pressure, *DBP* diastolic blood pressure, *TC* total cholesterol, *LDL-C* low-density lipoprotein cholesterol, *HDL-C* high-density lipoprotein cholesterol, *TG* triglyceride

^a^Compared between male and female subjects. *p < 0.05; **p < 0.01

### Comparison of demographic and baseline clinical characteristics of participants between low and high serum irisin group

Subjects were then divided into two groups based on the serum irisin level: a low irisin group (1.10–13.44 ng/ml) and a high irisin group (13.49–29.9 ng/ml). Subjects with high serum irisin levels had significantly higher HDL-C but lower FPG and were less likely to be a smoker than those with low serum irisin levels (Table 2). There were no significantly statistical differences in the age, active physical activity level, BMI, WC, 2hPG, TC, TG, LDL-C, SBP, DBP, fasting insulin, homeostatic model assessment-insulin resistance (HOMA-IR) or serum creatinine between these two groups.

**Table 2:** Comparison of demographic and baseline clinical characteristics of participants between low and high serum irisin groups

	Low irisin group	High irisin group	*p* value
N	96	95	
Sex (F/M)	66/30	75/20	0.109
Age (y)	57.2 ± 8.4	56.2 ± 8.4	0.394
Smoking (%)	31 (32.3%)	7 (7.4%)	≤ 0.001**
Active physical activity (%)	33 (34.4%)	42 (44.2%)	0.162
BMI (kg/m^2^)	27.6 ± 2.3	27.4 ± 2.5	0.672
WC (cm)	92.0 ± 7.4	91.2 ± 7.4	0.496
FPG (mmol/l)	5.8 (5.5, 6.1)	5.6 (5.4, 6.0)	0.025*
2hPG (mmol/l)	7.9 (6.7, 9.6)	7.6 (6.5, 9.0)	0.460
TC (mmol/l)	4.7 ± 1.1	4.6 ± 1.2	0.578
TG (mmol/l)	1.6 (1.1, 2.3)	1.5 (1.1, 2.2)	0.535
LDL-C (mmol/l)	2.7 ± 0.8	2.8 ± 0.7	0.395
HDL-C (mmol/l)	1.1 ± 0.3	1.3 ± 0.3	0.005**
SBP (mmHg)	121.8 ± 15.4	123.6 ± 19.4	0.489
DBP (mmHg)	78.7 ± 9.7	80.0 ± 10.6	0.385
Fasting insulin (mIU/l)	10.2 ± 3.4	10.3 ± 4.5	0.790
HOMA-IR	2.7 ± 1.0	2.7 ± 1.4	0.938
Serum creatinine (mmol/l)	69.1 ± 16.1	68.2 ± 13.6	0.652
Serum irisin (ng/ml)	10.96 (9.28, 12.29)	16.60 (14.33, 19.86)	≤ 0.001**

### Determination of association between serum irisin level and atherogenic factors

We next performed univariable and multivariable linear regression analysis to determine the association between serum irisin levels and atherogenic factors. As shown in Table 3, serum irisin levels were negatively associated with smoking (β = -3.443, p ≤ 0.001) and FPG (β = -1.205, p = 0.010), but were positively associated with HDL-C (β = 2.775, p = 0.013). After the multivariable linear regression analysis with adjustment for covariates, smoking was found to be negatively associated with serum irisin with statistical significance, while HDL-C was positively associated with irisin level with statistical significance. FPG was not significantly associated with serum irisin level.

**Table 3:** Determination of association between serum irisin levels and atherogenic factors

	Univariable linear regression		Multivariable linear regression	
	β	p value	β	p value
Age (y)	− 0.001	0.979		
Sex (male = 1, female = 2)	1.464	0.057		
Smoking (%)	− 3.434	≤ 0.001**	− 2.781	0.004**
FPG (mmol/l)	− 1.205	0.010**		
2hPG (mmol/l)	− 0.079	0.529		
TC (mmol/l)	− 0.114	0.691		
TG (mmol/l)^a^	− 0.235	0.244		
LDL-C (mmol/l)	− 0.004	0.992		
HDL-C (mmol/l)	2.775	0.013**	5.047	0.007**
SBP (mmHg)	0.015	0.438		
DBP (mmHg)	0.041	0.222		

SBP, systolic blood pressure; DBP, diastolic blood pressure; FPG, fasting plasma glucose; 2hPG, 2 h plasma glucose; HDL-C, high-density lipoprotein cholesterol; TC, total cholesterol; LDL-C, low-density lipoprotein cholesterol; TG, triglyceride

**p < 0.01

### Determination of association between serum irisin level and CVRFs

The present study mainly evaluated the correlation between serum irisin levels and four CVRFs: smoking, diabetes mellitus, hypertension and dyslipidemia. The prevalence of a single CVRF and the clustering of CVRFs is shown in Table 4. The high serum irisin group had a significantly lower prevalence of smoking, diabetes mellitus and dyslipidemia (7.4%, 8.4% and 51.6%, respectively) than the low serum irisin (32.3%, 18.8% and 71.9%, respectively). There was no significant difference in the prevalence of hypertension between these two groups. Additionally, the prevalence of the clustering of ≥ 1, ≥ 2, ≥ 3 CVRFs in the high irisin group were significantly lower than that of the low irisin group.

**Table 4:** Determination of association between serum irisin levels and CVRFs

	Lower irisin group	Upper irisin group	p value
*A single CVRF*			
Smoking	31 (32.3%)	7 (7.4%)	≤ 0.001**
Diabetes mellitus	18 (18.8%)	8 (8.4%)	0.037*
Hypertension	17 (17.7%)	19 (20.0%)	0.686
Dyslipidemia	69 (71.9%)	49 (51.6%)	0.004**
*Clustering of CVRFs*			
0	17 (17.7%)	36 (37.9%)	0.002**
≥ 1	79 (82.3%)	57 (62.1%)	0.002**
≥ 2	40 (41.7%)	18 (18.9%)	0.001**
≥ 3	16 (16.7%)	2 (2.1%)	0.001**

*p < 0.05; **p < 0.01

Table 5 shows the results of the logistic regression analysis for the correlation between the serum irisin level and CVRFs. Binary logistic regression was used to determine the correlation between the serum irisin level and one single CVRF with the ORs and 95% CIs, and showed that an increased serum irisin level was significantly associated with a reduced risk for smoking and dyslipidemia, in both unadjusted and adjusted models. Furthermore, increased serum irisin levels appeared to reduce the risk of diabetes mellitus in an unadjusted model. However, this association disappeared after adjustment for covariables. Multivariate logistic regression was also used to analyze the association between serum irisin levels and the clustering of CVRFs. Compared with the results of subjects with 0 CVRFs, the unadjusted ORs and 95%CIs of serum irisin for cases with 1, 2 and ≥ 3 CVRFs were 0.912 (0.840, 0.985), 0.800 (0.718, 0.892) and 0.753 (0.649, 0.875), respectively. After adjustment for age, gender, active physical activity and other CVRFs, the ORs and 95% CIs of the serum irisin level in cases with 1, 2 and ≥ 3 CVRFs were 0.910 (0.841, 0.985), 0.799 (0.715, 0.893) and 0.720 (0.643, 0.888), respectively. Thus, there was a negative association between the serum irisin level and the clustering number of CVRFs (p < 0.05).

**Table 5:** Determination of OR and 95% CI of the association of the serum irisin level with a single CVRF and clustering of CVRFs

	Unadjusted		Adjusted^a^	
	OR (95%CI)	*p* value	OR (95%CI)	*p* value
*A single CVRF*				
Smoking	0.813 (0.732, 0.904)	≤ 0.001**	0.829 (0.735, 0.934)	0.002**
Diabetes mellitus	0.887 (0.800, 0.984)	0.025*	0.939 (0.837, 1.053)	0.279
Hypertension	1.000 (0.926, 1.081)	0.994	1.021 (0.937, 1.113)	0.640
Dyslipidemia	0.862 (0.802, 0.926)	≤ 0.001**	0.868 (0.805, 0.935)	0.012*
*Clustering of CVRFs*				
0	1 (ref)		1 (ref)	
1	0.912 (0.840, 0.985)	0.019*	0.910 (0.841, 0.985)	0.020*
2	0.800(0.718, 0.892)	≤ 0.001**	0.799 (0.715, 0.893)	≤ 0.001**
3	0.753(0.649, 0.875)	≤ 0.001**	0.720 (0.643, 0.888)	0.001**
p for trend		≤ 0.001**		0.012*

Age, gender, active physical activity and other CVRFs are adjusted when estimate odds ratios (ORs) with 95% confidence intervals (CIs). *p < 0.05; **p < 0.01

## Discussion

The major finding from the present study was that serum irisin levels were negatively associated with the clustering of the major CVRFs among the Chinese overweight/obese population. To the best of our knowledge, this is the first study to investigate the relationship between serum irisin levels and the clustering of CVRFs among the Chinese adult overweight/obese populations.

Irisin was initially identified as a skeletal muscle-secreted factor. However, numerous studies have demonstrated that irisin is implicated in mediating the function of several tissues/organs and has been proposed as a biomarker of different diseases, including metabolic disease, sarcopenia and endothelial dysfunction [26–28]. In the cardiovascular field, previous studies that have investigated the adult population have mainly focused on the association of circulating irisin levels with a single CVRF, for example, with either diabetes mellitus or dyslipidemia. Serum irisin levels have been shown to be negatively associated with dyslipidemia, especially TC and LDL-C, among men [29]. However, irisin levels were found to be positively associated with HDL-C among patients with chronic kidney disease [30]. In the present study, we found that the risk for a single CVRF, such as smoking or dyslipidemia, was lower in the high irisin group than in the low irisin group, indicating an inverse correlation between serum irisin and smoking/dyslipidemia. Furthermore, we showed that there was a positive correlation between serum irisin and HDL-C, which is an acknowledged protective factor of cardiovascular disease. Notably, there was a negative association between serum irisin and smoking, offering another layer of evidence for a correlation between low serum irisin levels and cardiovascular diseases. Smoking may increase the circulating Interleukin-6 level which is a proinflammatory cytokine [31]. Inflammation may decrease the expression of *FNDC5* and cause the low level of serum irisin. Previously, Yan et al*.* revealed that the circulating irisin level was negatively associated with raised plasma glucose among the central obese population [17]. Additionally, a meta-analysis involving 17 cross-sectional and 6 case control studies also suggested that diabetes mellitus patients had lower circulating irisin levels than those of controls [18]. In the present study, we also found an inverse correlation between the serum irisin level and FPG and the prevalence of diabetes mellitus among the overweight/obese populations. However, this correlation disappeared after adjustment for gender, physical activity and other CVRFs. Thus, the exact correlation between the serum irisin level and diabetes mellitus needs to be further clarified.

Obesity is regarded as a risk factor for cardiovascular diseases [32]. Traditional risks factors for cardiovascular disease, such as diabetes mellitus, dyslipidemia and hyperuricemia are more likely to cluster in obese populations. The clustering of CVRFs greatly increases not only the incidence of cardiovascular disease but also the mortality compared with cases with an individual CVRF [33, 34]. In the present study, we found that the serum irisin level had an inverse association with the number of clustered CVRFs. We further revealed that a high serum irisin level was correlated to a decreased level of clustering of CVRFs among Chinese overweight/obese populations than a low serum irisin level. Consistent with the present study, several previous studies have shown that patients with coronary disease had lower serum irisin levels [12, 13]. It is well known that atherosclerosis underlies a variety of cardiovascular diseases, and that many CVRFs, including diabetes mellitus, dyslipidemia, smoking and hypertension, can promote the pathogenesis of atherosclerosis. We found that the serum irisin level had an independent relationship with smoking, dyslipidemia and the number of clustered CVRFs. Therefore, we conclude that the serum irisin level may act as a biomarker for the risk of cardiovascular diseases, at least in the overweight/obese population.

Previous studies have demonstrated the possible mechanism by which serum irisin prevents the development of atherosclerosis. In apoE-deficiency-induced atherosclerotic animal models, irisin administration decreases the atherosclerotic plaque area, as well as the inflammation and cell apoptosis observed in aortic tissues [35, 36]. In human umbilical vein endothelial cells, irisin decreases lipid-induced cell apoptosis and alleviates oxidative stress, which could prevent the occurrence of atherosclerosis [35–37]. Irisin also has effects on metabolism. For example, irisin impacts the metabolism of glucose and lipids [38–40]. Additionally, irisin induces cultured white adipocyte browning, resulting in an increase in energy expenditure and weight loss in high-fat-fed obese mice [38]. The administration of irisin in high-fat-fed obese mice reduces the expression of hepatic cholesterol genes [39] and hepatic gluconeogenesis [40]. In human hepatocellular carcinoma cells with an insulin-resistance state, irisin ameliorates glucose output and fat accumulation [41]. Taken together, these findings support a notion that irisin offers benefits for cardiovascular diseases.

The limitations of the present study should be acknowledged. First, the sample size of our study was small. Second, this was a cross-sectional study, which was limited to determine the association between serum irisin levels and a single or clustering of CVRFs, and/or cardiovascular diseases, but not the cause-effect correlation. Third, smoking, one of the main CVRFs in our present study, was defined by self-report, which might have introduced a certain level of bias to this study.

## Conclusions

In conclusion, our study was the first study to clarify the association between serum irisin levels and the clustering of CVRFs among the Chinese overweight/obese population. We demonstrated that high levels of serum irisin are negatively associated with smoking and dyslipidemia. We also showed that there was a negative correlation between serum irisin and the clustering of major CVRFs. Thus, we propose that serum irisin may serve as a biomarker of the pathogenesis of cardiovascular diseases. Prospective studies with larger populations are needed in the future to corroborate our findings and conclusions.

## Data Availability

The datasets generated and analyzed during the current study are not publicly available due to none of the data types requiring uploading to a public repository but are available from the corresponding author on reasonable request.

## Abbreviations

CVRFs: Cardiovascular risk factors
BMI: Body mass index
WC: Waist circumference
FPG: Fasting plasma glucose
2hPG: 2-Hour plasma glucose after the OGTT
HDL-C: High-density lipoprotein cholesterol
LDL-C: Low-density lipoprotein cholesterol
TC: Total cholesterol
TG: Triglyceride
HOMA-IR: Homeostatic model assessment -insulin resistance
DBP: Diastolic blood pressure
SBP: Systolic blood pressure

## References

[1] Fruh SM (2017). Obesity: risk factors, complications, and strategies for sustainable long-term weight management. J Am Assoc Nurse Pract.

[2] Collaborators GBDRF (2016). Global, regional, and national comparative risk assessment of 79 behavioural, environmental and occupational, and metabolic risks or clusters of risks, 1990–2015: a systematic analysis for the Global Burden of Disease Study 2015. Lancet.

[3] Huxley RR, Barzi F, Woo J, Giles G, Lam TH, Rahimi K (2014). A comparison of risk factors for mortality from heart failure in Asian and non-Asian populations: an overview of individual participant data from 32 prospective cohorts from the Asia-Pacific Region. BMC Cardiovasc Disord.

[4] Yang ZJ, Liu J, Ge JP, Chen L, Zhao ZG, Yang WY (2012). Prevalence of cardiovascular disease risk factor in the Chinese population: the 2007–2008 China National Diabetes and Metabolic Disorders Study. Eur Heart J.

[5] Bovet P, Arlabosse T, Viswanathan B, Myers G (2012). Association between obesity indices and cardiovascular risk factors in late adolescence in the Seychelles. BMC Pediatr.

[6] Fingeret M, Marques-Vidal P, Vollenweider P (2018). Incidence of type 2 diabetes, hypertension, and dyslipidemia in metabolically healthy obese and non-obese. Nutr Metab Cardiovasc Dis.

[7] Bostrom P, Wu J, Jedrychowski MP, Korde A, Ye L, Lo JC (2012). A PGC1-alpha-dependent myokine that drives brown-fat-like development of white fat and thermogenesis. Nature.

[8] Jedrychowski MP, Wrann CD, Paulo JA, Gerber KK, Szpyt J, Robinson MM (2015). Detection and quantitation of circulating human irisin by tandem mass spectrometry. Cell Metab.

[9] Varela-Rodriguez BM, Pena-Bello L, Juiz-Valina P, Vidal-Bretal B, Cordido F, Sangiao-Alvarellos S (2016). FNDC5 expression and circulating irisin levels are modified by diet and hormonal conditions in hypothalamus, adipose tissue and muscle. Sci Rep.

[10] Huh JY, Panagiotou G, Mougios V, Brinkoetter M, Vamvini MT, Schneider BE (2012). FNDC5 and irisin in humans: I. Predictors of circulating concentrations in serum and plasma and II. mRNA expression and circulating concentrations in response to weight loss and exercise. Metabolism.

[11] Hisamatsu T, Miura K, Arima H, Fujiyoshi A, Kadota A, Kadowaki S (2018). Relationship of serum irisin levels to prevalence and progression of coronary artery calcification: a prospective, population-based study. Int J Cardiol.

[12] Anastasilakis AD, Koulaxis D, Kefala N, Polyzos SA, Upadhyay J, Pagkalidou E (2017). Circulating irisin levels are lower in patients with either stable coronary artery disease (CAD) or myocardial infarction (MI) versus healthy controls, whereas follistatin and activin A levels are higher and can discriminate MI from CAD with similar to CK-MB accuracy. Metabolism.

[13] Deng W (2016). Association of serum irisin concentrations with presence and severity of coronary artery disease. Med Sci Monit.

[14] Hou N, Han F, Sun X (2015). The relationship between circulating irisin levels and endothelial function in lean and obese subjects. Clin Endocrinol (Oxf).

[15] Khorasani ZM, Bagheri RK, Yaghoubi MA, Chobkar S, Aghaee MA, Abbaszadegan MR (2019). The association between serum irisin levels and cardiovascular disease in diabetic patients. Diabetes Metab Syndr.

[16] Guo W, Zhang B, Wang X (2020). Lower irisin levels in coronary artery disease: a meta-analysis. Minerva Endocrinol.

[17] Yan B, Shi X, Zhang H, Pan L, Ma Z, Liu S (2014). Association of serum irisin with metabolic syndrome in obese Chinese adults. PLoS ONE..

[18] Qiu S, Cai X, Yin H, Zugel M, Sun Z, Steinacker JM (2016). Association between circulating irisin and insulin resistance in non-diabetic adults: a meta-analysis. Metabolism.

[19] Shen S, Gao R, Bei Y, Li J, Zhang H, Zhou Y (2017). Serum irisin predicts mortality risk in acute heart failure patients. Cell Physiol Biochem.

[20] Ning G, Reaction Study G (2012). Risk Evaluation of cAncers in Chinese diabeTic Individuals: a lONgitudinal (REACTION) study. J Diabetes..

[21] Zhou BF, Cooperative Meta-Analysis Group of the Working Group on Obesity in C (2002). Predictive values of body mass index and waist circumference for risk factors of certain related diseases in Chinese adults—study on optimal cut-off points of body mass index and waist circumference in Chinese adults. Biomed Environ Sci..

[22] Bi Y, Lu J, Wang W, Mu Y, Zhao J, Liu C (2014). Cohort profile: risk evaluation of cancers in Chinese diabetic individuals: a longitudinal (REACTION) study. J Diabetes.

[23] Perakakis N, Triantafyllou GA, Fernandez-Real JM, Huh JY, Park KH, Seufert J (2017). Physiology and role of irisin in glucose homeostasis. Nat Rev Endocrinol.

[24] Genuth S, Alberti KG, Bennett P, Buse J, Defronzo R, Kahn R (2003). Follow-up report on the diagnosis of diabetes mellitus. Diabetes Care.

[25] Chobanian AV, Bakris GL, Black HR, Cushman WC, Green LA, Izzo JL (2003). The seventh report of the Joint National Committee on prevention, detection, evaluation, and treatment of high blood pressure: the JNC 7 report. JAMA.

[26] Martinez Munoz IY, Camarillo Romero EDS, Garduno Garcia JJ (2018). Irisin a novel metabolic biomarker: present knowledge and future directions. Int J Endocrinol.

[27] Chang JS, Kim TH, Nguyen TT, Park KS, Kim N, Kong ID (2017). Circulating irisin levels as a predictive biomarker for sarcopenia: a cross-sectional community-based study. Geriatr Gerontol Int.

[28] El-Lebedy DH, Ibrahim AA, Ashmawy IO (2018). Novel adipokines vaspin and irisin as risk biomarkers for cardiovascular diseases in type 2 diabetes mellitus. Diabetes Metab Syndr.

[29] Oelmann S, Nauck M, Volzke H, Bahls M, Friedrich N (2016). Circulating irisin concentrations are associated with a favourable lipid profile in the general population. PLoS One..

[30] Wen MS, Wang CY, Lin SL, Hung KC (2013). Decrease in irisin in patients with chronic kidney disease. PLoS ONE.

[31] Kureya Y, Kanazawa H, Ijiri N, Tochino Y, Watanabe T, Asai K (2016). Down-regulation of soluble α-Klotho is associated with reduction in serum irisin levels in chronic obstructive pulmonary disease. Lung.

[32] Ortega FB, Lavie CJ, Blair SN (2016). Obesity and cardiovascular disease. Circ Res.

[33] Malik S, Wong ND, Franklin SS, Kamath TV, L'Italien GJ, Pio JR (2004). Impact of the metabolic syndrome on mortality from coronary heart disease, cardiovascular disease, and all causes in United States adults. Circulation.

[34] Povel CM, Beulens JW, van der Schouw YT, Dolle ME, Spijkerman AM, Verschuren WM (2013). Metabolic syndrome model definitions predicting type 2 diabetes and cardiovascular disease. Diabetes Care.

[35] Zhang Y, Mu Q, Zhou Z, Song H, Zhang Y, Wu F (2016). Protective effect of irisin on atherosclerosis via suppressing oxidized low density lipoprotein induced vascular inflammation and endothelial dysfunction. PLoS ONE..

[36] Lu J, Xiang G, Liu M, Mei W, Xiang L, Dong J (2015). Irisin protects against endothelial injury and ameliorates atherosclerosis in apolipoprotein E-Null diabetic mice. Atherosclerosis.

[37] Zhu D, Wang H, Zhang J, Zhang X, Xin C, Zhang F (2015). Irisin improves endothelial function in type 2 diabetes through reducing oxidative/nitrative stresses. J Mol Cell Cardiol.

[38] Zhang Y, Li R, Meng Y, Li S, Donelan W, Zhao Y (2014). Irisin stimulates browning of white adipocytes through mitogen-activated protein kinase p38 MAP kinase and ERK MAP kinase signaling. Diabetes.

[39] Tang H, Yu R, Liu S, Huwatibieke B, Li Z, Zhang W (2016). Irisin inhibits hepatic cholesterol synthesis via AMPK-SREBP2 signaling. EBioMedicine.

[40] Xin C, Liu J, Zhang J, Zhu D, Wang H, Xiong L (2016). Irisin improves fatty acid oxidation and glucose utilization in type 2 diabetes by regulating the AMPK signaling pathway. Int J Obes (Lond).

[41] So WY, Leung PS (2016). Irisin ameliorates hepatic glucose/lipid metabolism and enhances cell survival in insulin-resistant human HepG2 cells through adenosine monophosphate-activated protein kinase signaling. Int J Biochem Cell Biol.

